# Deep Q-Managed: a new framework for multi-objective deep reinforcement learning

**DOI:** 10.3389/frai.2025.1683323

**Published:** 2025-12-02

**Authors:** Richardson Menezes, Thiago Henrique Freire de Oliveira, Luiz Paulo de Souza Medeiros, Adrião Duarte Dória Neto

**Affiliations:** 1 Postgraduate Program in Electrical and Computer Engineering, Federal University of Rio Grande do Norte, Natal, Brazil; 2 Tractian Tecnologies Inc., São Paulo, Brazil; 3 Federal Institute of Rio Grande do Norte, Pau dos Ferros, Brazil

**Keywords:** Deep Q-Learning, Double Q-Learning, dueling networks, multiobjective reinforcement learning, machine learning

## Abstract

This paper introduces Deep Q-Managed, a novel multi-objective reinforcement leaning (MORL) algorithm designed to discover all policies within the Pareto Front. This approach enhances multi-objective optimization by integrating deep leaning techniques, including Double and Dueling Networks, to effectively mitigate the curve of dimensionality and overestimation bias. Deep Q-Managed demonstrates high proficiency in attaining non-dominated multi-objective policies across deterministic episodic environments, adapting to convex, concave, or mixed Pareto Front complexities. Experiments on traditional MORL benchmarks (Deep Sea Treasure, Bountiful Sea Treasure, and Modified Bountiful Sea Treasure) show it consistently achieves maximum hypervolume values (e.g., 1,155 for DST, 3,352 for BST, and 2,632 for MBST) and locates all Pareto Front points. While robust and versatile for practical applications in robotics, finance, and healthcare, this study's validation is currently confined to deterministic episodic settings, with stochastic environments reserved for future work.

## 1 Introduction

Reinforcement learning (RL) is a pivotal branch within the realm of machine learning, wherein an intelligent agent embarks on a quest to discern and execute optimal behaviors within an unfamiliar setting. This learning paradigm requires the agent to gradually discover the most advantageous actions to take within the myriad states presented by the environment. In the realm of RL, the agent adheres to a fundamental principle of learning through trial and error, meticulously investigating the consequences of various actions when confronted with specific environmental states. This process involves the analysis of scalar feedback signals, which are intricately linked to each action's consequences (Sutton and Barto, 2018). These feedback signals constitute the fundamental pillar of RL, guiding the agent's journey as it relentlessly strives to discern the optimal course of action for each potential scenario, adeptly refining its decision-making skills.

Nevertheless, a significant number of real-world challenges exceed the scope of a simple single feedback signal. Decision-making problems are often complex and require simultaneous optimization of multiple criteria or objectives, which are frequently conflicting. Prominent examples of such intricate challenges include multi-objective scheduling problems in workshops, which necessitate balancing conflicting criteria like production time, energy consumption, and product quality (Zhang et al., 2024). These objectives may share similarities or differences, but most of the time, they are caught up in a web of conflict, a scenario where boosting one objective's performance always results in a decrease in another's, and vice versa. Furthermore, there may exist multiple optimal solutions with varying priorities between objectives (Deb, 2008). This intricate interplay of objectives exemplifies the multifaceted nature of numerous real-world problems, necessitating nuanced resolutions that balance competing interests and priorities.

To address these complex decision-making scenarios, Multi-Objective Reinforcement Learning (MORL) has emerged, extending the RL paradigm to optimize a vector of objectives rather than a single reward signal. Various approaches have surfaced in the literature, all sharing the common goal of crafting specialized techniques for MORL. These techniques can broadly be categorized into two main groups. One group deals with the conversion of a Multi-objective Optimization (MOO) problem into a Single-objective Optimization (SOO) problem through a scalarizing function (Miettinen and Mäkelä, 2002). This approach typically yields a singular solution per run, making the algorithms that use it known as single-policy algorithms. Representative algorithms in this category include W-Learning (Humphrys, 1996), Modular Q-Learning (Karlsson, 1997), Scalarized MORL (Van Moffaert et al., 2013), W-Steering, and Q-Steering (Vamplew et al., 2015).

Another approach involves the search for multiple policies, which can be performed simultaneously or iteratively, one policy per run. The algorithms belonging to this category are referred to as multi-policy algorithms. When it comes to MOO and the pursuit of multiple policies, the most widely recognized and successful algorithms have historically been based on evolutionary algorithms, such as PESA (Corne et al., 2000), NSGA-II (Deb et al., 2002), and SPEA2 (Zitzler et al., 2001). Recent research has significantly advanced the integration of machine learning, particularly RL, with Multi-Objective Evolutionary Algorithms (MOEAs) to enhance their performance in complex optimization tasks, such as large-scale scheduling problems (Zhang et al., 2024). This integration is not only aimed at overcoming their respective limitations but also at creating a more comprehensive and powerful optimization framework, often saving computation time and potentially having lower overall sample-complexity by exploiting the fact that multiple policies need to be produced (Hayes et al., 2022).

Despite these advancements, many existing MORL approaches face significant limitations. A pervasive challenge is their inability to determine every conceivable optimal compromise solution, leaving portions of the objective space unexplored and underutilized. Furthermore, a significant subset of these MORL approaches suffers from limited generalizability, restricting their utility to particular application domains and curtailing their broader applicability.

Another critical hurdle is the curse of dimensionality, a challenge often faced by modern Q-Learning algorithms (Bellman, 1966). The number of states an agent must explore and evaluate increases exponentially as the dimensionality of the state space grows. Practically, as target problems grow more intricate, the state space can expand exponentially, rendering conventional Q-Learning approaches computationally impractical. This issue is of paramount importance, as it severely restricts the scalability and suitability of these algorithms in intricate, granular domains. Effectively mitigating the curse of dimensionality is critical for RL research, as it holds the key to unlocking the full potential of Q-Learning in tackling real-world, large-scale problems.

To overcome these challenges, this study introduces Deep Q-Managed, a new MORL algorithm that aims to discover all policies within the Pareto Front. This new approach builds upon the original Q-Managed framework, firstly presented in de Oliveira et al. (2021), by incorporating deep learning neural networks. This expansion enhances its potential for tackling more complex problems, empowering the algorithm to navigate and optimize in high-dimensional state spaces with greater efficiency and effectiveness.

Deep Q-Managed employs a hybrid MOO method, combining the use of a linear scalarizing function with an ϵ-constrained approach. A key aspect is the utilization of Deep Double Dueling Networks for the agent's learning. This approach not only aims to solve the curse of dimensionality that plagues traditional tabular RL algorithms (Poggio and Liao, 2018) but also capitalizes on the mitigation of overestimation bias and the accelerated learning convergence facilitated by the combination of these deep learning algorithms. The algorithm retains the main figure of the “manager” (de Oliveira et al., 2021), allowing agents to sequentially learn the set of policies that comprise the Pareto front for *a posteriori* choice, inheriting the capabilities, simplicity, and performance of single-policy algorithms. This capability to approximate and discover the entire Pareto Front is particularly crucial in highly complex and dynamic design spaces, such as those encountered in smart material optimization, where multi-objective RL frameworks like the Adaptive Pareto Optimization Model (APOM) are developed to approximate continuous Pareto Frontiers and handle multiple conflicting objectives like strength, flexibility, cost, and energy efficiency (Zou, 2025).

This methodology has the potential to generate a set of agents who possess specialized expertise in each of the policies, thereby forming the optimal solution set for a particular environment. The proposed algorithm achieves a desired characteristic of multi-policy algorithms to not be limited to a subset of the Pareto Front, and it achieves that with a competitive performance. Deep Q-Managed is capable of learning deterministic non-stationary policies and tackling problems where the region of optimal solutions may exhibit various shapes, such as convex, concave, or a combination of both. Furthermore, the algorithm is model-free, inheriting this property from its Q-Learning core. To verify this novel approach, extensive tests were conducted on conventional benchmarks utilized for MORL algorithms in environments where the Pareto Front is either convex, non-convex, or mixed, specifically the Deep Sea Treasure (DST), Bountiful Sea Treasure (BST), and Modified Bountiful Sea Treasure (MBST). While the deep learning techniques employed by the algorithm can handle non-episodic and stochastic environments, this paper solely addresses deterministic episodic problems that possess a clearly defined terminal state, with research into other problem types reserved for future work.

The remainder of this paper is structured as follows: Section 2 introduces key concepts of Deep RL and MORL and presents the proposed Deep Q-Managed algorithm in Section 2.4. Section 3 discusses the experimental results, and Section 4 provides the conclusion and outlines future research directions.

## 2 Materials and methods

### 2.1 Deep reinforcement learning

Deep Reinforcement Learning (DRL) is an artificial intelligence sub-field that integrates deep neural networks (DNNs) with reinforcement learning (RL) to address complex decision-making in high-dimensional environments. Traditional RL algorithms frequently encounter difficulties with vast state spaces and intricate tasks (Goodfellow et al., 2016). DRL effectively overcomes these challenges by leveraging DNNs as powerful function approximators, enabling agents to represent and learn from extensive and complex data.

This integration is critical for frameworks like Deep Q-Managed, directly supporting its ability to tackle multi-objective problems. DNNs allow agents to learn complex mappings between states, actions, and rewards, facilitating generalization from observed experiences and decision-making in unexplored state regions (LeCun et al., 2015). By processing high-dimensional input data, DRL agents can extract meaningful features and intricate patterns directly from the environment, which is essential for tasks involving perception, representation, and decision-making in real-world scenarios, including multi-objective scheduling problems (Zhang et al., 2024).

Crucially, DRL techniques directly support the Deep Q-Managed framework by mitigating the curse of dimensionality that limits traditional tabular RL algorithms (Bellman, 1966; Poggio and Liao, 2018). This capability is instrumental in expanding Deep Q-Managed's potential for tackling more complex multi-objective problems and navigating high-dimensional state spaces with greater efficiency and effectiveness. Furthermore, DRL algorithms address the fundamental challenge of balancing exploration (trying new actions) and exploitation (using gathered information to maximize rewards), a key aspect in the successful discovery of optimal policies.

### 2.2 Multiobjective deep reinforcement learning

Multiobjective reinforcement learning (MORL) is a branch of reinforcement learning that involves multiple, competing goals. An agent learns to take actions in an environment to maximize a single reward signal in a traditional reinforcement learning problem. In many real-world situations, however, an agent may have multiple objectives that conflict with one another. A robot, for example, may have to navigate a crowded environment while avoiding collisions and conserving energy. In these cases, a single objective function cannot capture all the agent's goals.

MORL algorithms are designed to handle these types of multi-objective optimization problems by considering multiple conflicting objectives at the same time. MORL algorithms use multiple reward signals to represent different objectives, rather than a single reward signal. The algorithms then learn a policy that attempts to balance these competing goals and find a compromise.

One of the most difficult aspects of MORL is determining the objectives and their relative importance. In some cases, the objectives are clear and well-defined; in others, they are hazy or uncertain. MORL algorithms must be able to deal with uncertainty and adapt to changing goals.

Before proceeding to the formal definition of MORL, it is necessary to define a MOO. In summary, a MOO can be defined as follows:


$$\begin{array}{l} \max F\left(\text{X}\right)=\left[f_{1}\left(\text{X}\right),f_{2}\left(\text{X}\right),\ldots ,f_{m}\left(\text{X}\right)\right]\\ s.t.g_{l}\left(\text{X}\right)\leq 0\;l=1,\ldots ,L \end{array}$$ (1)

where *m* denotes the number of objective functions, *L* the number of constraint functions of the problem, and *X* = [*x*_1_, …, *x*_*N*_] the vector of variables to be optimized (Deb and Kalyanmoy, 2001; Back, 1996).

In contrast to single objective optimization problems, where the reward is a scalar value, MORL provides the agent with a reward vector of the same size as the number of objectives because each vector position is a reward associated with a specific objective, among several that the learning agent must optimize, as shown in the reward formulation seen in Equation 2.


$$\begin{array}{l} R\left(s,a\right)=\left[R_{1}\left(s,a\right),R_{2}\left(s,a\right),\ldots ,R_{m}\left(s,a\right)\right] \end{array}$$ (2)

Similarly, a vector formulation of the state value function and state-action value function can be structured, as shown in Equations 3, 4, respectively.


$$\begin{array}{l} {\text{V}}^{\pi }\left(s\right)={𝔼}_{\pi }\left[\overset{\infty }{\underset{k=0}{\sum }}\gamma^{k}{\text{r}}_{t+k+1}|s_{t}=s\right] \end{array}$$ (3)

$$\begin{array}{l} {\text{Q}}^{\pi }\left(s,a\right)={𝔼}_{\pi }\left[\overset{\infty }{\underset{k=0}{\sum }}\gamma^{k}{\text{r}}_{t+k+1}|s_{t}=s,a_{t}=a\right] \end{array}$$ (4)

The optimal vector state-action can then be defined according to Equation 5.


$$\begin{array}{l} {\text{Q}}^{*}\left(s,a\right)=\text{R}\left(s,a\right)+𝔼\left[\gamma \underset{a^{\prime }}{\max}{\text{Q}}^{*}\left(s^{\prime },a^{\prime }\right)\right] \end{array}$$ (5)

Given that the environment has multiple objectives at the same time, different optimal policies can be found, with the difference being related to the priority given to each. Various optimality criteria can be used to find optimal policies, since it is a MOO, the concept of Pareto dominance is commonly used (Back, 1996; Knowles and Corne, 2002).

Traditionally, the Pareto dominance relation (Pareto, 1964) is used to compare two solutions. We can use it to determine whether a solution is superior or inferior to others. This is formulated in the MORL context by the following definitions:

**Definition 2.1**. Given two policies π∈**Π** and π′∈**Π**, policy π is said to dominate policy π′ (π′≺π) if the following criteria are met:


$$\begin{array}{c} \pi^{\prime }≺\pi \Leftrightarrow \exists j:{\text{V}}_{j}^{\pi }\left(s\right)>{\text{V}}_{j}^{\pi^{\prime }}\left(s\right)\wedge \\ \forall i\neq j:{\text{V}}_{i}^{\pi }\left(s\right)≮{\text{V}}_{i}^{\pi^{\prime }}\left(s\right),\forall s\in S \end{array}$$ (6)

**V**^π^(*s*) is strictly better than **V**^π^′(*s*) on at least one objective, and **V**^π^(*s*) isn't strictly worse than **V**^π^′(*s*) on no other objective across all states.

** Definition 2.2**. If no other policy π′∈**Π** dominates policy π∈**Π**, π it is said to be Pareto optimal:


$$\begin{array}{l} \pi \text{ is Pareto optimal}\Leftrightarrow \forall \pi^{\prime }\in \Pi :\pi ≮\pi^{\prime } \end{array}$$ (7)

These formulations result in a set of optimal solutions for the Pareto front (Deb and Sundar, 2006).

Overall, MORL is an effective tool for resolving complex, real-world problems with multiple, competing objectives. It enables agents to learn and adapt in complex environments whilst also balancing multiple goals.

### 2.3 Combined use of Double and Dueling Networks in reinforcement learning

Reinforcement Learning (RL) algorithms have demonstrated significant promise in solving intricate decision-making issues. Two significant advancements in RL, namely Double Q-Learning and Dueling Networks, have garnered significant attention due to their capacity to enhance learning stability and enhance performance. In this section, we explore the combined use of Double and Dueling Networks and highlight their benefits in the context of RL applications.

#### 2.3.1 Double Q-Learning

The Double Q-Learning technique is an enhancement of conventional Q-Learning algorithms that addresses the issue of overestimation bias (Van Hasselt et al., 2016). By decoupling the action selection and value estimation processes, Double Q-Learning reduces overoptimistic value estimations. It maintains two sets of Q-values: an online network for action selection and a target network for unbiased value estimation. This decoupling improves learning stability and results in more accurate value estimates.

In traditional Q-Learning, the action-value function *Q*(*s, a*) is updated using the Bellman equation (Sutton and Barto, 2018):


$$\begin{array}{l} \begin{array}{c} Q\left(s,a\right)\leftarrow Q\left(s,a\right)+\alpha \cdot \left(r+\gamma \cdot \underset{a^{\prime }}{\max}Q\left(s^{\prime },a^{\prime }\right)-Q\left(s,a\right)\right) \end{array} \end{array}$$ (8)

Where *Q*(*s, a*) represents the Q-value of taking action *a* in state *s*, α is the learning rate, *r* is the immediate reward, γ is the discount factor, *s*′ is the next state, and *a*′ is the next action.

The update equation involves selecting the action *a*′ that will maximize the Q-value in the next state *s*′. This may however lead to an overestimation of the Q-values because the same Q-value is used for both action selection and value estimation.

This overestimation bias is addressed by Double Q-Learning, which decouples the action selection and value estimation processes. Instead of relying solely on a singular set of Q-values, Double Q-Learning maintains two distinct sets of Q-values, commonly referred to as the online network and the target network.

The online network is used for action selection, where the action with the highest Q-value estimate is chosen. The target network, on the other hand, is periodically updated using the online network's Q-values. This changes the equation for updating to:


$$\begin{array}{l} Q\left(s,a;\theta_{k}\right)\leftarrow Q\left(s,a;\theta_{k}\right)+\alpha \cdot (r+\gamma \cdot Q(s^{\prime },\underset{a^{\prime }}{arg max}Q(s^{\prime },a^{\prime };\\ \theta_{k}^{-}))-Q(s,a;\theta_{k})) \end{array}$$ (9)

Where *Q*′(*s*′, *a*′) represents the Q-value estimate from the target network. By using the target network to estimate the Q-values for action selection, Double Q-Learning reduces the overestimation bias and provides more accurate value estimates. The Target-Network parameters $\theta_{k}^{-}$ are only updated every *C* iterations with the Q-network parameters θ_*k*_ and are held fixed between updates (Van Hasselt et al., 2016).

#### 2.3.2 Dueling networks

The Dueling Networks approach aims to address the challenge of efficiently estimating state values and action advantages (Wang et al., 2016). By separating the estimation of state values and action advantages, Dueling Networks provide a more effective and focused learning process. The network architecture comprises two streams. One stream estimates the state value function, which denotes the value of being in a particular state, while the other stream estimates the action advantages, which denote the distinctions between actions in that state. This separation facilitates a more accurate representation of state values and action advantages, resulting in enhanced learning and decision-making.

Considering a reinforcement learning problem involving a discrete action space and a set of states, the objective is to acquire an optimal policy that maximizes the anticipated return over time. The state-value function, *V*(*s*), denotes the value of being in state *s*, whereas the action-value function, *Q*(*s, a*), denotes the value of taking action *a* in state *s*.

The Dueling Network architecture consists of two distinct components: the state value function, *V*(*s*), and the action advantage function, *A*(*s, a*). This method of decomposition facilitates a more effective assessment of state values and the advantages of actions. The state value function estimates the value of being in a specific state, whereas the action advantage function estimates the disparities in values among actions in that state.

The formal mathematical formulation of Dueling Networks can be expressed as follows, with θ^1^ representing the weights of the network before the two distinct components, θ^2^ representing the weights of the advantage function neural network approximator, and θ^3^ representing the weights of the value function neural network:


$$\begin{array}{l} Q\left(s,a;\theta^{1},\theta^{2},\theta^{3}\right)=V\left(s;\theta^{1},\theta^{3}\right)\\ +\left(A\left(s,a;\theta^{1},\theta^{2}\right)-\frac{1}{\left|A\right|}\underset{a^{\prime }\in A}{\sum }A\left(s,a^{\prime };\theta^{1},\theta^{2}\right)\right) \end{array}$$ (10)

Where *Q*(*s, a*) represents the action-value function, which is the sum of the state value function *V*(*s*) and the action advantage function *A*(*s, a*). *V*(*s*) represents the state value function, which estimates the value of being in state *s*. *A*(*s, a*) represents the action advantage function, which estimates the differences in values between actions in state *s*. The term $\frac{1}{\left|A\right|}\underset{a}{\sum }A\left(s,a^{\prime }\right)$ represents the mean advantage value, which ensures the identifiability of the action advantages by subtracting the mean advantage across all actions (Wang et al., 2016).

The Dueling Network design utilizes a loss function that encompasses both the state value loss and the advantage loss during training. The state value loss is intended to reduce the discrepancy between estimated and desired state values. The advantage loss tries to reduce the difference between the estimated and target action advantages.

By separating the estimation of state values from action benefits, this design provides for more focused learning and improved generalization across states and actions. It enables the agent to accurately estimate the value of staying in a specific state while evaluating the variations in values across actions. This separation improves learning and results in improved performance on reinforcement learning tasks.

#### 2.3.3 Combined use and benefits

The Deep Q-Managed algorithm critically integrates the combined principles of Double Q-Learning and Dueling Networks, as it shows remarkable performance enhancements in diverse RL applications (Wang et al., 2016; Mnih et al., 2015; Dabney et al., 2018), referred to as Deep Double Dueling networks, as the foundation for its agent's learning mechanism. This integrated approach is a cornerstone of Deep Q-Managed's methodological contributions, specifically designed to address key challenges in MORL and ensure both clarity and reproducibility (Tamar et al., 2016).

The primary methodological contribution of integrating these techniques into Deep Q-Managed is the effective mitigation of overestimation bias (a benefit of Double Q-Learning) and accelerated, more efficient learning (a benefit of Dueling Networks). This combined framework enables Deep Q-Managed agents to make informed decisions without overly optimistic estimations and facilitates better generalization across diverse states and actions. This robust approach is instrumental in addressing the well-known curse of dimensionality that often limits traditional tabular RL algorithms (Poggio and Liao, 2018).

By leveraging Deep Double Dueling networks, Deep Q-Managed expands its potential for tackling complex multi-objective problems in high-dimensional state spaces with greater efficiency and effectiveness. Furthermore, Deep Q-Managed is a model-free algorithm, inheriting this property from its core Q-Learning framework. The agent learns iterative sets of deterministic non-stationary policies within episodic environments to discover all Pareto-optimal policies.

Within the Deep Q-Managed framework, the agent's learning process, as detailed in Algorithm 1, involves iteratively updating its neural network parameters based on experiences gathered during environmental exploration. The use of Deep Double Dueling networks means that the neural network architecture for approximating Q-values incorporates both the dual network structure for target value calculation and the split stream architecture for state value and action advantage estimation.

Algorithm 1 Deep Q-Managed.

 1:  Initialize neural network parameters θ
 2:  Initialize replay buffer *D* with capacity *N*
 3:  for episode = 1 to *M* **do**
 4:   Initialize environment state *s*_0_
 5:   repeat
 6:   With probability ϵ, select a random action *a*_*t*_
 7:   Otherwise, select action *a*_*t*_ = argmax_*a*_*Q*(*s*_*t*_, *a*; θ)
 8:   Execute action *a*_*t*_, observe reward *r*_*t*_ and next state *s*_*t*+1_
 9:   if next state *s*_*t*+1_ is a final state already blocked **then**
 10:   Unblock the solution if it is a shorter path
 11:   end **if**
 12:   Store transition (*s*_*t*_, *a*_*t*_, *r*_*t*_, *s*_*t*+1_) in *D*
 13:   Sample batch of transitions from *D*
 14:   Set target $y_{i}=r_{i}+\gamma \cdot \underset{a^{\prime }}{\max}Q\left(s_{i+1},a^{\prime };\theta^{-}\right)$
 15:   Update parameters θ by minimizing MSE loss
 16:   Every *C* steps, update target network: θ^−^←θ
 17:   Set *s*_*t*_←*s*_*t*+1_
 18:   until *s*_*t*_ is a final state
 19:   Stores trajectory identifier as a hash string
 20:   if convergence is detected as repetition of hashs **then**
 21:   Reset ϵ and some agent parameters
 22:   Block solution
 23:   end **if**
 24:  end **for**

### 2.4 Deep Q-Managed

In this section, the Deep Q-Managed algorithm is further described. This algorithm is an enhanced version of the Q-Managed approach to MORL, and it incorporates certain concepts of methods derived from MOO, such as epsilon-constraint and scalarization technique. The name of the algorithm is given due to the combination of Deep Q-Learning technics in its multi-objective version and the fact that there is a figure to manage the behavior of the algorithm, a manager, proposed in de Oliveira et al. (2021).

Deep Q-Managed is capable of learning deterministic non-stationary policies and tackling problems where the region of optimal solutions may exhibit certain shapes, such as convex, concave, or a combination of both. Furthermore, the algorithm is model-free, since the core of the algorithm derives from Q-Learning and inherits this property. Lastly, it is noteworthy to mention that despite the ability of the deep learning techniques employed by the algorithm to handle non-episodic and stochastic environments, this paper solely addresses deterministic episodic problems that possess a clearly defined terminal state. The research into the application of this new technique to these other types of problems is being left for a future paper.

**Figure 1:**
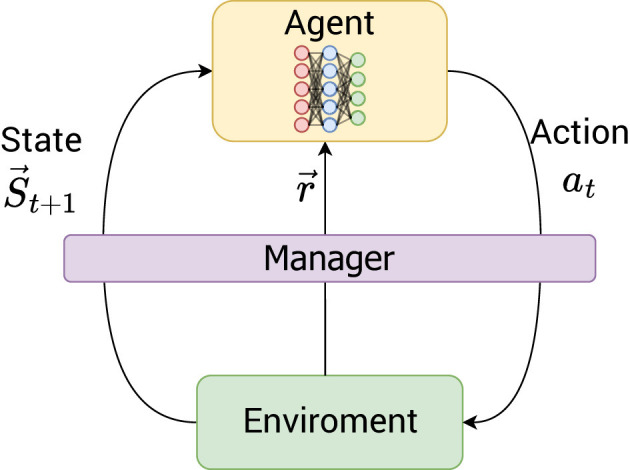
Deep Q-Managed framework: manager-environment-agent interaction for multi-objective reinforcement learning. This diagram illustrates the interplay of the agent, environment, and manager within the Deep Q-Managed algorithm. The agent learns optimal behaviors by interacting with the environment (actions, states, and rewards). The manager acts as a crucial supervisory component, observing the environment and strategically intervening in the agent's learning.

The manager concept is employed to gain a thorough understanding of the interactions between the learning agent and the objective environment, as well as to intervene in the actions being undertaken; its contribution is illustrated in Figure 1. The primary functions of this instrument are to assist the learning agent in identifying the whole set of optimal policies.

As the guide for the learning agent, the manager is responsible for observing what happens in the environment. Thus, whenever a learning agent performs an action that results in a terminal state, the manager observes the current policy's value function to compare it to the best value found and stored up to that point.

The learning of a given policy implies a terminal state linked to it. The convergence criterion will be discussed in Section 3. Whenever the agent reaches a terminal state associated with a previously acquired policy, the manager conducts a comparison of the value function associated with the present policy. This is intended to evaluate whether the path to the final state associated with this policy is superior to the one currently stored. The manager updates the policy if there is a successful comparison, to reflect the better path learned in the current episode. Otherwise, the manager modifies the learning agent's action to avoid unnecessary repetition, changing it to one that moves it to a random neighboring state, except for the terminal state. This strategy allows the agent to learn other policies and offers the possibility of changing to a superior one if one is found.

It is noteworthy that upon the learning agent's convergence to a particular policy, the randomness probability parameter is reset to 0.3, thereby enabling the agent to randomly explore the environment searching for policies for the undiscovered final states.

Given the difficulty of identifying the impact of one objective on another, it is recognized that the trade-off between objectives is extremely complicated, even though there is no guarantee that the solution will be isomorphic to the parameterization used (Das and Dennis, 1997). The algorithm contours the problem using a synthetic objective function, where no objective has a different degree of importance than any other. This can be characterized as a linear scalarizing function:


$$\begin{array}{l} \forall o\in \left[1,m\right]:w_{o}=\frac{1}{m}\overset{m}{\underset{o=1}{\sum }}w_{o}=1 \end{array}$$ (11)

This approach relieves the decision-maker of the concern about which objective to prioritize and if there is a solution corresponding to the parameterization chosen *a priori*.

The algorithm terminates when the number of converged policies equals the number of constraints, as each constraint represents a terminal state. This keeps the agent from learning all the policies and still continuing to explore the environment.

The algorithm is based on the extensive Q-learning framework, which has demonstrated remarkable efficacy in single-objective reinforcement learning tasks. Deep Q-learning involves the training of a neural network to approximate the Q-values of state-action pairs, which represent the anticipated cumulative reward of taking a specific action in a particular state. Through learning these Q-values, the agent can make informed decisions about which actions to take in different situations to maximize its cumulative reward.

The proposed Algorithm 1 involves the agent learning a set of policies for traversing environments with multiple objectives by iteratively updating its neural network parameters based on the experiences acquired during exploration. The fundamental innovation lies in the incorporation of a management mechanism that dynamically adjusts the agent's behavior to encourage exploration and guarantee convergence toward optimal solutions.

The management system works by periodically assessing the agent's progress and detecting alignment with optimal policies. Upon detection of convergence, the management mechanism intervenes by resetting agent parameters and blocking the encountered solution path. This enables the agent to explore alternate routes and continuously refine its policies to attain a more comprehensive coverage of the solution space.

In addition to managing the exploration-exploitation trade-off and overseeing the agent's learning process, the manager also plays a critical role in optimizing the agents' search for optimal solutions. During the main loop of learning, when the agent attempts to navigate to a final state associated with an already converged solution, the manager intervenes to assess the quality of the current solution. If the manager determines that the current solution is superior to those previously discovered, it initiates a process to unblock the path to that solution. By unblocking the path, the agent is granted the opportunity to explore this potentially better route, thus enabling it to potentially discover more efficient and effective solutions. This dynamic intervention by the manager ensures that the agent's learning process remains adaptive and responsive to evolving conditions, ultimately enhancing the algorithm's ability to identify optimal policies in complex environments.

#### 2.4.1 Experimental setup

The proposed Deep Q-Managed algorithm was tested on the traditional MORL benchmark and its variations, Deep Sea Treasure (DST) (Vamplew et al., 2008) and Bountiful Sea Treasure (BST) (Van Moffaert et al., 2014), to assess its performance and capabilities. In addition to the benchmark tests already mentioned, the algorithm was also evaluated on a variation of the BST proposed in (de Oliveira et al., 2021), the Modified Bountiful Sea Treasure (MBST). This section describes the benchmarks used, how the tests were carried out, and how they were evaluated.

**Figure 2:**
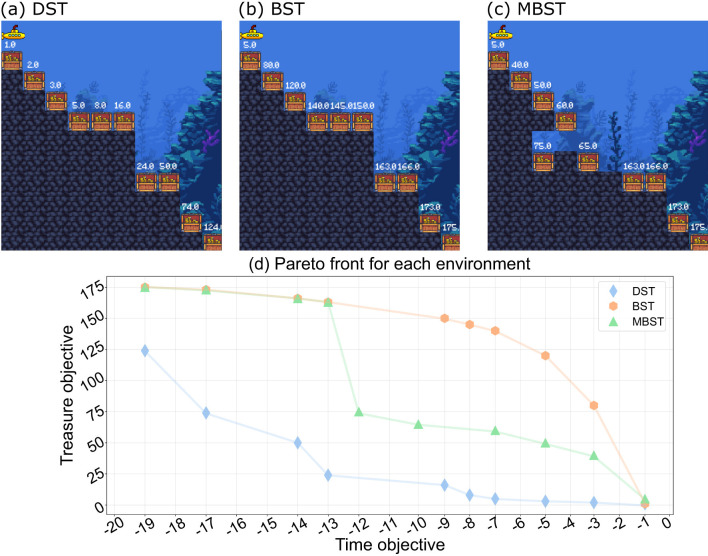
Benchmark environments used to evaluate the Deep Q-Managed algorithm. **(a)** Deep Sea Treasure (DST), **(b)** Bountiful Sea Treasure (BST), and **(c)** Modified Bountiful Sea Treasure (MBST). In each grid-based environment, the agent controls a submarine starting from the upper-left corner and navigates to treasure locations with varying rewards. DST features a concave Pareto Front, BST a convex Pareto Front, and MBST a mixed shape with local concavities. **(d)** Illustrates the corresponding Pareto Fronts for each environment, which serve as ground truth for assessing the ability of Deep Q-Managed to identify all optimal trade-off solutions.

Deep Sea Treasure (DST), shown in Figure 2a is an episodic deterministic problem in which a learning agent controls a vessel on an underwater expedition searching for treasure. The environment is made up of a grid of 11 rows and 10 columns containing 10 treasure locations, each with a different value assigned, with the lowest treasure value located closest to the starting point and the highest value positioned farthest away, implying that the value increases with distance from the source.

Each episode begins with the submarine in the upper-left corner and ends when the learning agent comes across any of the ten treasure locations, regardless of value, or completes 1,000 actions. The agent can navigate the environment by moving in any of the four cardinal directions: (I) up, (II) right, (III) down, or (IV) left. Any movement that causes the agent to leave the grid is disregarded, keeping it in the same position.

In this environment, the learning agent has two goals: to minimize the time needed to reach the treasure location and to maximize the value of the treasure. The reward vector is composed of two elements. The first of them is a penalty of –1 for each action performed. The second is the value of the treasure, which is 0 until the agent moves to a location containing a treasure. The DST's Pareto Front is globally concave with local concavities.

The only difference between the BST, shown in Figure 2b, and the DST is in the treasure values, which give the Pareto Front a globally convex shape. Everything remains the same as the DST in terms of environment structure, learning agent movements, and reward system.

Lastly, there is the variation of the BST, the MBST, pictured in Figure 2c. The changes in the environment in this variation are the treasure site location, the best time situation to reach it, and some treasure values. With the new treasure position, the learning agent must now enter a 'hole' in the middle of two different locations, which makes learning difficult because it can find one of the two places in the middle to pass through and end the episode. For this, variation the Pareto Front has a concavity in the middle dividing the curve into two convex parts.

## 3 Results

In this section, we present the outcomes of our investigation into the integration of Double and Dueling Networks in the Deep Q-Managed approach to MORL. The purpose of this study was to investigate the performance enhancements and benefits achieved by integrating these two key advancements into the Q-Managed algorithm.

It provides a thorough analysis of the results obtained from our experiments. We demonstrate the remarkable performance improvements achieved through the combined use of Double and Dueling Networks in the analyzed RL tasks. Specifically, we present empirical evidence for accelerated learning convergence, improved sample efficiency, and enhanced decision-making capabilities of RL agents. Since overestimation bias is reduced, informed and reliable decision-making can be achieved, while the separation of state values and action advantages provides a more focused learning process.

The parameters utilized for conducting the experiments are described in Table 1. The choice of values for hyperparameters was empirical, and achieved through a series of tests, which resulted in the same quality of policies found, with only a small range of episodes needed for convergence.

**Table 1:** Hyperparameters used in the Deep Q-Managed experiments.

**Parameter**	**Description**	**Value**
α	Learning rate	1·10^−4^
γ	Reward discounting	0.99
Initial ϵ	Randomness	0.9
Final ϵ		0.1
Restored ϵ		0.3
τ	ϵ decay constant	3·10^−3^
*w*_*o*_∀*o*∈[1, *m*]	Objectives weights	1/m
*m*	Number of objectives	2
*z*_1_	Reference time objective	-25
*z*_2_	Reference treasure objective	0

Values were selected to balance learning stability and convergence speed across environments.

A stringent convergence criterion was established to ensure a thorough evaluation and robust learning of the agent. This criterion introduces a distinct and crucial requirement: the agent must replicate the identical path to a treasure state a predetermined number of times. This criterion distinguishes our approach by setting a convergence delta of zero, thus rendering it exceptionally meticulous in gauging the agent's learning.

The convergence criterion serves as a litmus test for the agent's proficiency in achieving the final states in a consistent and reliable manner. By mandating the repetition of the same path toward a treasure state, we emphasize the significance of not only discovering a successful trajectory once but mastering it. This stringent criteria not only measures the agents' ability to learn, but also requires an elevated level of accuracy in their actions.

In contrast to convergence criteria that solely focus on attaining a predetermined reward or achieving consistent performance, this methodology does not allow for approximation or variability. The agent is compelled precisely to replicate its successful routes, thereby ensuring a level of comprehension and proficiency that is uncommon in convergence assessments.

The hypervolume indicator, which is provided by the learning agent after each experiment, serves as the metric utilized to evaluate the algorithms' performance (Vamplew et al., 2011). The resulting hypervolume of policies is then compared to the corresponding hypervolume of the best-case scenario represented by the Pareto Front.

The selection of the corresponding reference points for the hypervolume indicator was motivated by the desire to establish a fair comparison between the proposed algorithm and those cited in the literature. There are different methodologies for selecting reference points; however, this specification must consider the Pareto front's shape in addition to other aspects to be evaluated, such as performance or solution distribution. According to Ishibuchi et al. (2018), the methodology replicated in this work, with reference points much worse than the nadir point, is adequate for the type of problem and what is being evaluated.

The proposed methodology for the Deep Q-Managed algorithm has proven to be efficacious and efficient in identifying all optimal policies for the Pareto Front, and this is applicable to all environments. In terms of efficiency, the algorithm behaved similarly in all tests and learned the optimal policy, successfully repeating the paths to all final state, that being considered a conversion to an optimal policy for that state.

**Figure 3:**
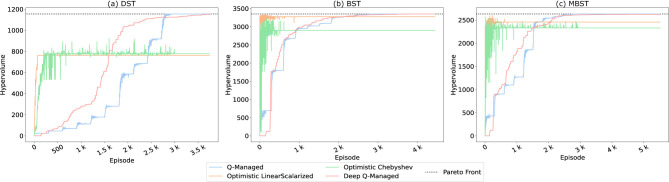
Average hypervolume values obtained by Deep Q-Managed across 20 independent runs for the three benchmark environments: **(a)** Deep Sea Treasure (DST), **(b)** Bountiful Sea Treasure (BST), and **(c)** Modified Bountiful Sea Treasure (MBST). The x-axis represents the number of training episodes, while the y-axis shows the hypervolume relative to the true Pareto Front. Different curves correspond to varying convergence thresholds (100–300 repetitions). Higher thresholds impose stricter convergence criteria, requiring the agent to replicate paths more consistently before confirming a policy. Results show that Deep Q-Managed consistently reaches the maximum hypervolume in all environments, though convergence in MBST is slower and more computationally demanding.

Figure 3 provides a visualization of the experimentation, showcasing the hypervolume averages across 20 independent runs. This serves as a testament to the performance of the proposed approach, as it consistently attains maximum hypervolume values across all three target environments. Specifically, in the DST environment, the hypervolume peaks at 1,155. In the BST environment, it soars to 3,352. Finally, in the MBST environment, the maximum hypervolume is 2,632.

Furthermore, Figure 3 demonstrates the exploration of different threshold values as a parameter for verifying conversion to a treasure state. These thresholds play a pivotal role in assessing the agent's convergence rigor. A higher threshold value signifies a more stringent evaluation criterion, demanding that the agent precisely replicate the same path to a treasure state a higher number of times. This adaptability in threshold values underscores the versatility of our approach, capable of accommodating diverse challenges and learning scenarios.

Notably, our experimental results underscore the robustness and adaptability of the proposed approach. Across all threshold values and environment combinations, our method consistently identifies and encompasses all points in the Pareto Front. This exceptional capability highlights the approach's versatility and its ability to excel in a multitude of environments.

The consistent inclusion of all Pareto Front points across different scenarios underscores the adaptability and robustness of our approach, positioning it as a contender in the realm of MORL methodologies.

The Deep Q-Managed approach has demonstrated its capabilities in the analyzed environments. The robust learning capabilities, efficient convergence, and adaptability of this approach have established it as a contender in the field. In the present investigation, our objective is not solely to introduce the most recent advancement of the Deep Q-Managed algorithm. But also to meticulously evaluate its advancements and performance in comparison to its predecessor and other pertinent algorithms from the existing literature.

Our primary objective is to elucidate the enhancements and innovations that have been incorporated into the Deep Q-Managed approach. By conducting this comprehensive analysis, we aim to provide a comprehensive understanding of the algorithm's strengths, thereby illuminating its potential for broader applications.

To accomplish this evaluation, we have devised a comparative framework. This framework entails a side-by-side examination of the latest Deep Q-Managed algorithm with the previous version of the algorithm proposed in de Oliveira et al. (2021). Moreover, we derive insights from two additional noteworthy algorithms proposed in Van Moffaert (2016), namely Linear Scalarized and Chebyshev Scalarized, which were devised and tested on the same benchmark analyzed in this work. The optimistic prefix was added to the algorithm's name to serve as an indication that the results presented herein were exclusively derived from the test phase, with any outcomes from the training phase being intentionally omitted or discarded. This multi-faceted comparison allows us to assess the progress made by the Deep Q-Managed approach and provides valuable context for its achievements.

Our proposed approach is evaluated against these relevant methodologies, not only in terms of learning efficiency, but also in terms of its adaptability to diverse tasks. The rigorous evaluation of the algorithm across a suite of environments demonstrates its versatility and potential.

**Figure 4:**
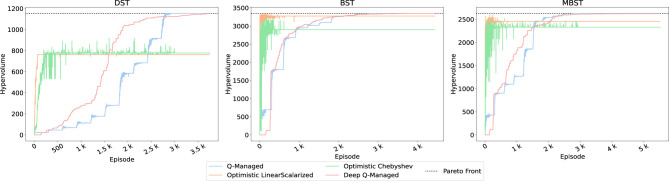
Comparative performance of Deep Q-Managed against three reference algorithms: (i) Q-Managed (de Oliveira et al., 2021), (ii) Linear Scalarized, and (iii) Chebyshev Scalarized (Van Moffaert, 2016). Results are reported on the three benchmark environments (DST, BST, MBST) in terms of achieved hypervolume relative to the true Pareto Front. Both Deep Q-Managed and Q-Managed consistently identify all Pareto-optimal points across environments, while scalarized methods locate only subsets of the Front (particularly underperforming in DST). Deep Q-Managed requires more episodes to discover the full Front but achieves faster identification of initial solutions and higher robustness across environments.

To accomplish this comparison, Figure 4 presents a comparative evaluation of the proposed Deep Q-Managed algorithm, alongside the three other pertinent strategies. The purpose of this evaluation is to emphasize the capabilities and characteristics of each algorithm in navigating the test scenarios.

From this analysis, it is striking that only the Deep Q-Managed and the Q-Managed approaches consistently found all the points in the Pareto front across all environments. This demonstrates their flexibility and robustness in addressing the multi-objective nature of the tasks. Despite not being able to find the full Pareto front, the Linear Scalarized and Chebyshev approaches demonstrated commendable performance in the BST and MBST environments. It is noteworthy that their final hypervolume in DST falls short of expectations, achieving only half of the maximum value. This disparity in performance across environments underscores the unique challenges posed by each setting and the varying adaptability of the algorithms.

An examination of the algorithms that encountered the full Pareto front reveals an interesting trade-off between the Deep Q-Managed and the other approaches. While Deep Q-Managed required more episodes to find all points within the Pareto front, it exhibited a notable advantage in discovering most of the initial points more quickly. The algorithms' efficiency in identifying the initial points, especially those associated with shorter paths or not located in plateaus, demonstrates efficient exploration of rewarding regions.

The remarkable capability of Deep Q-Managed, alongside Q-Managed, to consistently locate all points in the Pareto front across diverse environments merits highlighting. The disparity in performance observed in DST, BST, and MBST underscores the intricate and contextually dependent nature of these tasks. Furthermore, the tradeoff between exploration and exploitation efficiency in Deep Q-Managed underscores its unique approach to multi-objective problem-solving. This provides a nuanced perspective on algorithmic performance and provides valuable insight into their strengths and adaptability across challenging environments.

**Figure 5:**
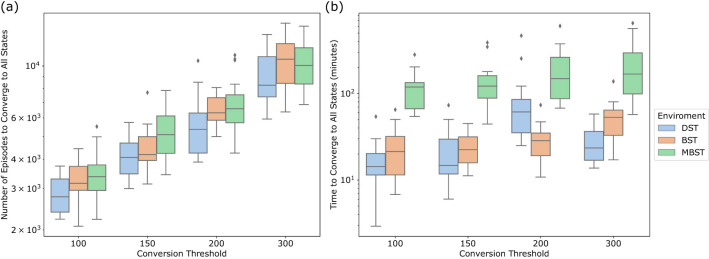
Convergence analysis of Deep Q-Managed across different conversion thresholds (100–300 repetitions) for the three benchmark environments: **(a)** distribution of the number of episodes required for the agent to converge to all treasure states, and **(b)** distribution of the processing time (minutes) needed to achieve full convergence. Results are averaged over 20 independent runs. DST consistently requires fewer episodes and less computation, reflecting its simpler structure, whereas MBST demands substantially more episodes and processing time due to its higher complexity and local concavities. These results illustrate the trade-off between stricter convergence criteria, which ensure policy reliability, and the additional computational resources required.

To further examine the tradeoffs inherent in the Deep Q-Managed's performance, Figure 5a examines the number of episodes required by the Deep Q-Managed algorithm to reach all final states, spanning 20 independent executions in all tested environments, each separated by distinct conversion thresholds. The resulting investigation illuminates the inherent stochastic properties of the Deep Q-Managed strategy and emphasizes its flexibility and effectiveness, particularly in scenarios involving lower conversion thresholds.

It is noteworthy that the Deep Q-Managed algorithm exhibits a total of convergence episodes that are comparable to the 3,000 episodes observed in Q-Managed. In certain instances, the algorithm can achieve significantly faster convergence, indicating its capacity for rapid learning and decision-making in environments with less stringent convergence criteria. This enhanced performance in scenarios that necessitates precise and swift convergence exemplifies the algorithm's adaptability and its potential to excel.

To build on the analysis total number of episodes needed for total convergence, Figure 5b delves into the processing time required by the Deep Q-Managed algorithm to complete each environment across various threshold configurations. Since the duration of the operations is entirely dependent on the hardware being employed, it is pertinent to mention that, for the experiments presented here, a system comprising an Intel i7-8565U CPU, 16 GB of RAM, and an NVIDIA GeForce MX150 GPU was utilized.

The box plots depicted in Figure 5b highlight the diverse processing times employed by the Deep Q-Managed algorithm when tackling diverse environments and threshold combinations. It is evident that the DST environment consistently requires less processing time for completion. This outcome is in accordance with expectations, as DST also necessitated a lesser number of total episodes, thereby demanding fewer computational resources.

Conversely, the MBST environment consistently exhibits a notably higher processing time requirement. This observation emphasizes the complexity and resource-intensive nature of this environment, compared to others. The extended processing times in MBST demonstrate the algorithm's dedication and computational effort needed to navigate the multi-objective landscape effectively.

The varying processing times underscore the flexibility of the Deep Q-Managed algorithm in handling diverse environments and threshold configurations. Although processing times may vary, the algorithm demonstrates its ability to efficiently allocate computational resources to address the distinct challenges posed by each environment. This ability to adapt is particularly advantageous when the significance of computational efficiency is paramount.

The analysis presented here showed that the Deep Q-Managed algorithm is a multifaceted approach to MORL, characterized by a set of tradeoffs and distinct advantages that set it apart from other methodologies. These characteristics contribute to its adaptability, efficiency, and innovative contributions.

One notable tradeoff observed in Deep Q-Managed pertains to its stochastic nature regarding convergence. Across different environments and conversion thresholds, the number of episodes required for completion is variable. While this might entail longer convergence times stochastically in some cases, it also underscores the algorithms' capacity to adapt to different challenges. This adaptability is in accordance with the dynamic nature of reinforcement learning tasks, where precise convergence times can exhibit significant variations.

Another thing that was found is that the algorithm processing time varies a lot between environments and threshold configurations. The complexity of the environment and the stringent nature of conversion criteria are some of the factors that cause this variability. While Deep Q-Managed may require longer processing times in certain instances, this attribute highlights its capacity to effectively allocate computational resources, ensuring that challenging tasks are tackled with the appropriate computational resources.

However, despite these tradeoffs, Deep Q-Managed boasts several notable advantages. One of its greatest strengths lies in its ability to explore and exploit points in the Pareto front. It consistently identifies all points within the Pareto front, demonstrating its adeptness in multi-objective problem-solving, a vital skill in practical applications where decisions often involve balancing multiple objectives.

Furthermore, Deep Q-Managed achieves competitive convergence, especially in scenarios with lower conversion thresholds. This efficiency demonstrates its adaptability to learning and making decisions in environments that require precise and rapid convergence. Its ability to perform well across diverse environments underscores its robustness and versatility.

Another asset is the proposed approaches resource allocation capabilities. It utilizes computational resources efficiently, tailoring its efforts to meet the specific challenges posed by each environment.

This presents a compelling mix of tradeoffs and advantages, which reflect the dynamic and multifaceted nature of reinforcement learning environments. The stochastically and fluctuating processing times of the algorithm are balanced by its competitive convergence, adaptive resource allocation, and exceptional multi-objective capabilities. These strengths position Deep Q-Managed as an approach that has the potential to excel in demanding, real-world applications.

## 4 Conclusion

This paper introduced Deep Q-Managed, a multi-objective reinforcement learning framework that combines Double Q-learning and Dueling Network architectures to address overestimation bias and the curse of dimensionality. Extending the original Q-Managed method with deep neural networks, the approach enables systematic discovery of Pareto-optimal policies across environments with convex, concave, and mixed Pareto Fronts.

Across all benchmarks, Deep Q-Managed consistently attained the maximum hypervolume and successfully identified every point of the Pareto Front. Compared to scalarized baselines, it demonstrated superior coverage and robustness, while retaining competitive convergence rates relative to the original Q-Managed framework. Notably, convergence in DST and BST was achieved efficiently, whereas MBST required longer training and higher computational effort due to its more complex Pareto structure. This highlights a characteristic trade-off of the method: broader policy discovery at the expense of increased computational demand in challenging environments.

The present study focused on deterministic episodic environments and used averaged hypervolume indicators as the primary measure of performance. Extending this analysis to stochastic or continuous domains, incorporating statistical tests, and exploring parallel multi-agent learning represent natural directions to strengthen generality and efficiency. Looking forward, extending validation to continuous control tasks [e.g., MuJoCo (Todorov et al., 2012)], incorporating formal statistical analyses, and exploring parallel multi-agent training are promising directions to strengthen both scalability and robustness.

Deep Q-Managed thus offers a flexible and reproducible framework for multi-objective reinforcement learning. With open-source code available at https://github.com/xarmison/deep_q_managed, we aim to encourage further validation and application in real-world domains such as robotics, finance, and healthcare.

In conclusion, Deep Q-Managed represents an advancement in the field of MORL. It exhibits adaptability and robustness, navigating environments with convex, concave, or hybrid Pareto fronts. Its prowess at locating and exploiting points in the Pareto front demonstrates its aptitude for tackling multiple objectives. The core concept behind Deep Q-Managed is to provide a versatile, effective, and cooperative framework for tackling the diverse obstacles in multi-objective environments.

## Data Availability

The datasets presented in this study can be found in online repositories. The names of the repository/repositories and accession number(s) can be found below: https://github.com/xarmison/deep_q_managed.
